# Thermal radiation effects on nanofluid flow over a vertical cone in the presence of pressure work

**DOI:** 10.1038/s41598-025-10554-5

**Published:** 2025-08-04

**Authors:** Mohamed Fathy, Emad A. Sayed

**Affiliations:** 1 Basic and Applied Science Department, College of Engineering and Technology, Arab Academy for Science, Technology and Maritime Transport, Cairo, Egypt; 2 Department of Physics and Engineering Mathematics, Faculty of Engineering-Mattaria, Helwan University, Cairo, Egypt

**Keywords:** Thermal radiation, Vertical truncated cone, Laminar boundary layer, Pressure work, Nanofluid, Legendre collocation method, Applied mathematics, Fluid dynamics

## Abstract

This study examines the effect of thermal radiation on nanofluid flow and heat transfer over a truncated cone in the presence of pressure work, a problem critical for thermal management and industrial cooling systems. Using similarity transformations, the governing equations are converted into coupled nonlinear partial differential equations and solved numerically via the Legendre collocation method. It gives a high degree of consistency between the proposed numerical solutions and the results previously reported under specific cases. The Prandtl number, pressure work parameter, radiation parameter, and nanoparticle volume fraction all have a major impact on flow and thermal behavior, according to the main results. Nanofluids enhance the transfer of heat by 10–40% when compared to pure fluid, cooling speeds up, and surface strength and hardness improve. Also, the kinds of nanofluid and the parameters related to the volume percentage of nanoparticles are crucial in determining the flow behavior. The surface mechanical properties are advanced by using 10% nanoparticle nanofluid rather than 5%. It has been discovered that the strength and hardness of the surface will enhance with an increase in the pressure work parameter when employing Cu-water nanofluid, but they will decrease with an increase in the thermal radiation parameter values. The novelty of this work lies in the application of the Legendre collocation method to this problem, along with new quantitative insights into how pressure work and radiation interact with nanofluids, providing practical guidelines for optimizing thermal and mechanical performance in industrial systems.

**Supplementary Information:**

The online version contains supplementary material available at 10.1038/s41598-025-10554-5.

## Introduction

Convection flow research is essential to many scientific and industrial fields, such as sophisticated thermal management systems, nuclear reactor cooling, and star and planet formation. For engineering solutions to be optimized, it is crucial to comprehend temperature distribution and heat transfer, especially when pressure work and free convection are involved. Even though natural convection along vertical surfaces has been thoroughly investigated, configurations involving thermal radiation, non-uniform surface temperatures, and nanofluids are still being researched because of their potential to greatly improve heat transfer efficiency in energy applications and cooling systems^1–11^.

The foundation for comprehending these systems was established by early studies of convection flows across conical geometries. General laws for analogous solutions on isothermal axisymmetric shapes, such as vertical cones, were established by Merk and Prins^12,13^. Roy^14^ expanded the work to high Prandtl number fluids, whereas Hering and Grosh^15^ investigated laminar free convection from non-isothermal cones at low Prandtl numbers. In more recent studies, laminar free convection from vertical circular cones with non-uniform surface temperatures and pressure work was studied by Alim et al.^16^ and Alam et al.^17^. These investigations’ relevance to sophisticated heat transport situations was limited, though, because they did not completely address thermal radiation or nanofluids.

By examining natural convection boundary layer flow over a truncated cone embedded in a porous medium saturated by a nanofluid with constant wall temperature and nanoparticle volume percent, Cheng^18^ made a substantial contribution to the field of truncated cone research. In this study, thermophoresis and Brownian motion were included. It was discovered that although lowering the buoyancy ratio or Lewis number raises the local Nusselt number, raising these parameters decreases it. It did not, however, include heat radiation or pressure work, which are essential for solar energy systems and electronic cooling, for example. Noghrehabadi et al.^19^ studied the natural-convection flow of nanofluids over vertical cone embedded in non-darcy porous media. Sayed and Fathy^20^ studied the upward cone flow of nanofluids and the effects of thermal radiation and heat generation on heat transfer. A powerful, consistent transverse magnetic field perpendicular to the cone surface is applied to the flow.

Recent research has begun to explore thermal radiation in nanofluid flows. For instance, Ragulkumar et al.^21^ investigate the MHD water-based nanofluid flow via an upright cone. The heat and mass flux pattern is used in this mathematical model to examine MHD, viscous dissipation, radiation, chemical reactions and suction/injection processes. Vinutha et al.^22^ examined the influence of a magnetic field on nanofluid passing through a cone and wedge with mass and heat transmission.. While this study advances the understanding of nanofluid behavior under thermal radiation, it focuses on vertical cones rather than truncated cones and does not address pressure work, leaving a gap in the literature for truncated cone-specific configurations.

Despite these advancements, the combined effects of pressure work, thermal radiation, and nanofluids on convection flow over a truncated cone remain underexplored. Studies like Cheng^18^, and Noghrehabadi et al.^19^ provide valuable insights into truncated cone configurations but do not address the interplay of all three factors pressure work, thermal radiation, and nanofluids. Similarly, Sayed and Fath^20^ highlight the importance of thermal radiation but focus on vertical cones without considering pressure work or truncated cone geometries.

This study introduces key innovations, including a comprehensive analysis of pressure work’s influence on free convection in a nanofluid, the incorporation of thermal radiation effects using a nonlinear temperature model, and a comparative assessment of different nanofluid types to optimize heat transfer performance. Notably, the Legendre collocation method, which is a high-precision numerical approach not previously applied to this class of problems, is implemented to solve the governing equations, providing superior accuracy in capturing boundary layer dynamics and heat transfer characteristics. By addressing these gaps, our work provides new insights into advanced thermal systems, contributing to applications in nuclear cooling, aerospace engineering, and nanofluid-based heat exchanger design.

## The governing equations and mathematical formulation

**Fig. 1:**
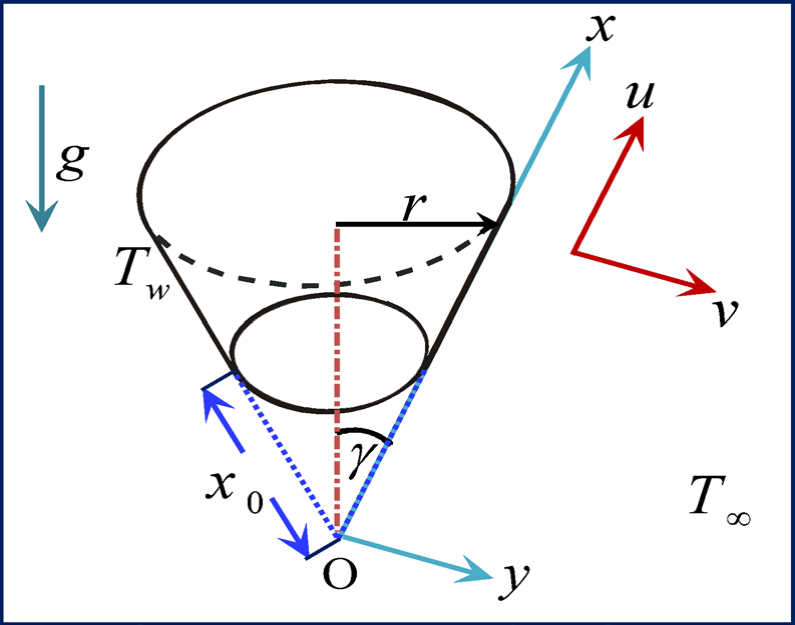
Coordinate system and physical model.

Laminar free convection flow in two dimensions via a truncated cone will be examined in a steady state. Given that $y$ is the coordinate normal to the surface of the cone and $x$ represents the position along the cone’s surface as measured from the origin, the physical coordinates $(x, y)$ are selected so that the coordinates’ origin is at the cone’s vertex. ${x}_{0}$ is a measurement of the truncated cone’s leading edge’s distance from the origin. Figure 1 displays the flow setup and coordinate system. Copper Cu, silver Ag, and titanium TiO_2_ are among the many nanoparticles present in the water-based nanofluid. The assumption is that there is no slip between the nanoparticles and the base fluid and that they are in thermal equilibrium.

Using a vertical cone, the following boundary layer governing equations^16,23^:

1 $$\frac{\partial }{\partial x}(ur)+\frac{\partial }{\partial y}(\nu r)=0,$$

2 $${\rho }_{nf}\left(u\frac{\partial u}{\partial x}+\nu \frac{\partial u}{\partial y}\right)={\mu }_{nf}\frac{{\partial }^{2}u}{\partial {y}^{2}}+{\text{g}}{ \left(\rho \beta \right)}_{nf}\text{cos}\gamma (T-{T}_{\infty }),$$

3 $${\left(\rho {C}_{p}\right)}_{nf}\left(u\frac{\partial T}{\partial x}+v\frac{\partial T}{\partial y}\right)={k}_{nf}\frac{{\partial }^{2}T}{\partial {y}^{2}}+{\beta }_{nf}Tu\frac{\partial p}{\partial x}-\frac{\partial {q}_{r}}{\partial y},$$

**Fig. 2:**
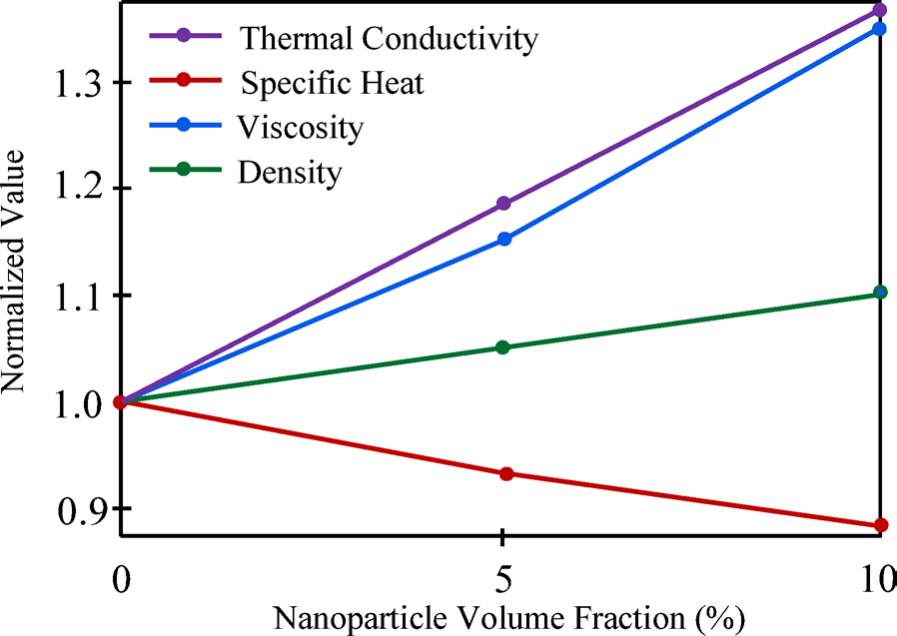
Normalized thermophysical properties of Cu–water nanofluid the versus nanoparticles concentration.

The boundary conditions are provided by

4 $$u=0,\nu =0,T={T}_{w}\text{ at }y=0,u=0,T={T}_{\infty }\text{ as } y\to \infty ,$$

where $u$ and $\nu$ represent the fluid velocity components in the $x$ and $y$ directions, respectively, and $r$ is the cone’s radius. In this case, ${\mu }_{nf}$, ${\rho }_{nf}$, ${\beta }_{nf}$ and ${C}_{p}$ stand for the nanofluid’s dynamic viscosity, density, coefficient of thermal expansion coefficient of nanofluid, and specific heat at constant pressure, respectively. ${\text{g}}$ is the acceleration caused by gravity, $T$ is the temperature of the nanofluid, ${T}_{\infty }$ is the ambient temperature, ${T}_{w}$ is the temperature of the cone surface, ${k}_{nf}$ is the nanofluid’s thermal conductivity, and $\gamma$ is the cone apex half angle. Table 1 provides the nanoparticles’ thermophysical properties^20,24,25^. Table 2 introduces the thermophysical properties of nanofluids: Cu, Ag, TiO_2_ and water at 5% nanoparticle volume fraction (25 °C)^26–29^. A Cu–water nanofluid’s normalized thermo-physical characteristics are shown in Fig. 2 as the concentration of nanoparticles rises from 0 to 10%. There is a noticeable improvement in thermal conductivity, which suggests improved heat transmission capacity. Additionally, viscosity rises, which may increase the need for pumping power and flow resistance. With the addition of more Cu nanoparticles, density increases linearly and the specific heat decreases.

**Table 1:** Water and nanoparticle thermophysical properties.

	$\rho \,\,\left( {kg/m^{3} } \right)$	$C_{p} \,\left( {J/k{\text{g}}K} \right)$	$k\,\,\left( {W/mK} \right)$	$\beta \times 10^{5} \,\left( {1/K} \right)$
${\text{H}}_{2}{\text{O}}$	997.1	4179	0.6130	21.0
${\text{Cu}}$	8933	385.0	401.00	1.67
${\text{Ag}}$	10,500	235.0	429.00	1.89
${\text{TiO}}_{2}$	4250	686.2	8.9538	0.90

**Table 2:** Thermophysical properties of nanofluids: Cu, Ag, TiO_2_ and water at 5% nanoparticle volume fraction (25 °C).

Property	Base Fluid (Water)	Cu-Water Nanofluid	Ag-Water Nanofluid	TiO_2_-Water Nanofluid
Thermal Conductivity $\left( {W/mK} \right)$	0.613	0.74	0.85	0.71
Density $\left( {kg/m^{3} } \right)$	997	1385	1475	1162
Specific Heat $\left( {J/k{\text{g}}\;K} \right)$	4179	3650	3550	3850

We can define the hydrostatic pressure under external conditions as $\partial p/\partial x=-{\rho }_{nf} \, {\text{g}}$, and using the radiation Rosseland approximation^30^, the radiative heat flux, ${q}_{r}$, may be expressed simply as

5 $${q}_{r}=-\frac{4{\sigma }^{*}}{3{\alpha }^{*}}\frac{\partial {T}^{4}}{\partial y},$$

where ${\alpha }^{*}$ and ${\sigma }^{*}$ stand for the mean absorption coefficient and Stefan-Boltzmann constant, respectively. To state the term ${T}^{4}$ to be as a linear function of temperature, we assume that the temperature gradients within the flow are sufficiently large. Therefore, by ignoring higher-order terms and extending ${T}^{4}$ in a Taylor series around ${T}_{\infty }$, it becomes

6 $${T}^{4}\cong 4{T}_{\infty }^{3}T-3{T}_{\infty }^{4}.$$

Equations (5) and (6) are used to transform the energy Eq. (3) into

7 $${\left(\rho {C}_{p}\right)}_{nf}\left(u\frac{\partial T}{\partial x}+\nu \frac{\partial T}{\partial y}\right)={k}_{nf}\frac{{\partial }^{2}T}{\partial {y}^{2}}+{\beta }_{nf}Tu\frac{\partial p}{\partial x}+\frac{16\sigma {T}_{\infty }^{3}}{3{\alpha }^{*}}\frac{{\partial }^{2}T}{\partial {y}^{2}}.$$

The following are the characteristics of the nanofluid.

8 $$\begin{array}{c}{R}_{1}=\displaystyle\frac{{\mu }_{nf}}{{\mu }_{f}}={\left(1-\phi \right)}^{-2.5},{R}_{2}=\displaystyle\frac{{\rho }_{nf}}{{\rho }_{f}}=(1-\phi )+\phi\displaystyle\frac{{\rho }_{s}}{{\rho }_{f}},{R}_{3}=\frac{{(\rho \beta )}_{nf}}{{(\rho \beta )}_{f}}=(1-\phi )+\phi \displaystyle\frac{{(\rho \beta )}_{s}}{{(\rho \beta )}_{f}},\\ {R}_{4}=\displaystyle\frac{{(\rho {C}_{p})}_{nf}}{{(\rho {C}_{p})}_{f}}=(1-\phi )+\phi \displaystyle\frac{{(\rho {C}_{p})}_{s}}{{(\rho {C}_{p})}_{f}},{R}_{5}=\displaystyle\frac{{k}_{nf}}{{k}_{f}}=\displaystyle\frac{{k}_{s}+2{k}_{f}-2\phi ({k}_{f}-{k}_{s})}{{k}_{s}+2{k}_{f}+\phi ({k}_{f}-{k}_{s})},\end{array}$$

It should be noted that when $\phi =0$, the study drops to those of a viscous or ordinary fluid, where $\phi$ is the volume percentage of nanoparticles. The following kinds of similarity hold for the Eqs. (1), (2) and (7) under condition Eq. (4):

$$\xi =\frac{{x}^{*}}{{x}_{0}}=\frac{x-{x}_{0}}{{x}_{0}},\eta =\frac{y}{{x}^{*}}{\left(G{r}_{{x}^{*}}\right)}^{1/4},\psi ={\upsilon }_{f}r{\left(G{r}_{{x}^{*}}\right)}^{1/4}f\left(\xi ,\eta \right),$$

9 $$T-{T}_{\infty }=\left({T}_{w}-{T}_{\infty }\right)\theta \left(\eta \right),r=x\text{sin}\gamma ,$$

In the boundary layer region, the functions $f\text{(}\xi \text{, }\eta \text{)}$ and $\theta {(}\xi {,}\eta {)}$ represent the stream function and the temperature function of the fluid, respectively, in dimensionless form, while $G{r}_{{x}^{*}}={\text{g}} \, {\beta }_{f}\text{cos}\gamma ({T}_{w}-{T}_{\infty }){\left({x}^{*}\right)}^{3}/{{\upsilon }_{f}}^{2}$ is the local Grashof number and $\eta$ is the pseudo-similarity variable. The definitions of $u$ and $\nu$ that fulfill Eq. (1) are $u=\frac{1}{r}\frac{\partial \psi }{\partial y}$ and $\nu = - \frac{1}{r}\frac{\partial \psi }{{\partial x}}$, if $\psi$ is the stream function. The preceding nonlinear partial differential equations system were produced by replacing the transformations provided in Eq. (9) into Eqs. (2), (4), and (7):

10 $$\frac{{R_{1} }}{{R_{2} }}f^{\prime \prime \prime } + \left[ {\frac{\xi }{1 + \xi } + \frac{3}{4}} \right]ff^{\prime \prime } - \frac{1}{2}f^{\prime 2} + \frac{{R_{3} }}{{R_{2} }}\theta = \xi \left[ {f^{\prime } \frac{{\partial f^{\prime } }}{\partial \xi } - f^{\prime \prime } \frac{\partial f}{{\partial \xi }}} \right],$$

11 $$\frac{1}{\text{Pr}}\left[\frac{{R}_{5}+{N}_{R}}{{R}_{4}}\right]{\theta }^{\prime\prime}+\left[\frac{\xi }{1+\xi }+\frac{3}{4}\right]f{\theta }^{\prime}-\varepsilon {f}^{\prime}\theta =\xi \left[{f}^{\prime}\frac{\partial \theta }{\partial \xi }-{\theta }^{\prime}\frac{\partial f}{\partial \xi }\right],$$

subject to the following boundary conditions:

12 $$f(\xi ,0)={f}^{\prime}(\xi ,0)=0,\theta (\xi ,0)=1,{f}^{\prime}(\xi ,\eta )=0,\theta (\xi ,\eta )=0\text{ as }\eta \to \infty .$$

Here, the differential with regard to $\eta$ is indicated by the primes, $\text{Pr}={\upsilon }_{f}\text{/}{\alpha }_{f}$ indicates the Prandtl number, ${N}_{R}=16\sigma {{T}_{\infty }}^{3}/3{\alpha }^{*}{k}_{f}$ for radiation parameter, and $\varepsilon ={\text{g}} {\beta }_{nf} {x}^{*}/{({C}_{p})}_{nf}$ for pressure work parameter, which Gebhart^31^ was the first to employ.

The two most important components of the flow from an engineering perspective are the skin friction coefficient and the Nusselt number, which stand for heat transfer rate and surface shear stress, respectively. The mechanical properties of the surface are directly impacted by these characteristics throughout the heat treatment process. For example, when the heat transfer rate, heat flux, increases at the material surface, the cooling speed arises. Therefore, it enhances the surface’s shear strength and hardness while decreasing its ductility, which raises the risk of surface cracking. The surface shear stress ${\tau }_{w}$, the skin friction coefficient ${C}_{f}$, surface heat flux ${q}_{w},$ and the Nusselt number ${Nu}_{{x}^{*}}$ are given as:

$${\tau }_{w}={\mu }_{nf}{\left(\frac{\partial u}{\partial y}\right)}_{y=0}={\mu }_{nf}\left[\frac{{\upsilon }_{f}{\left(G{r}_{{x}^{*}}\right)}^{3/4}}{{\left({x}^{*}\right)}^{2}}{f}^{\prime\prime }\left(\xi ,0\right)\right],$$

13 $${q}_{w}=-{k}_{nf}{\left(\frac{\partial T}{\partial y}\right)}_{y=0}=-{k}_{nf}\left[\frac{\left({T}_{w}-{T}_{\infty }\right){\left(G{r}_{{x}^{*}}\right)}^{1/4}}{{x}^{*}}{\theta }^{\prime}\left(\xi ,0\right)\right],$$

14 $${C}_{f}=\frac{2{\tau }_{w}}{{\rho }_{f}{U}^{2}}=\frac{2{R}_{1}{f}^{\prime\prime }(\xi ,0)}{{(G{r}_{{x}^{*}})}^{1/4}}, N{u}_{{x}^{*}}=\frac{ {x}^{*}{q}_{w}}{{k}_{f}({T}_{w}-{T}_{\infty })}={\left(G{r}_{{x}^{*}}\right)}^{1/4}\left({R}_{5}\right)\left(-{\theta }^{\prime}\left(\xi ,0\right)\right),$$

where $U={\upsilon }_{f}{(G{r}_{{x}^{*}})}^{1/2}/{x}^{*}$ is the reference velocity.

## Legendre polynomials

The first kind of Legendre polynomial, ${P}_{n}(x)$, of degree $n$ is generated when the Legendre differential equation is solved. It is defined by^32^:

15 $$P_{n} \left( x \right) = \frac{1}{{2^{n} }}\mathop \sum \limits_{\ell = 0}^{\lfloor{\frac{n}{2}}\rfloor} \left( { - 1} \right)^{\ell } \left( {\begin{array}{*{20}c} n \\ \ell \\ \end{array} } \right)\left( {\begin{array}{*{20}c} {2n - 2\ell} \\ n \\ \end{array} } \right)x^{n - 2\ell } = 2^{n} \mathop \sum \limits_{\ell = 0}^{n} \left( {\begin{array}{*{20}c} n \\ \ell \\ \end{array} } \right)\left( {\begin{array}{*{20}c} {\frac{n + \ell - 1}{2}} \\ n \\ \end{array} } \right)x^{\ell },$$

where $x\in \left[-1,1\right]$ with recurrence relations^33^

16 $${P}_{n+1}^{\prime}\left(x\right)-{P}_{n-1}^{\prime}\left(x\right)=\left(2n+1\right){P}_{n}\left(x\right),$$

17 $$x{P}_{n}^{\prime}(x)-{P}_{n-1}^{\prime}(x)=n{P}_{n}(x)$$

### **Lemma 1**

^33,34^ *Let* $u(\zeta ,\chi )\in {H}^{\kappa }(-{1,1})\cap {H}^{{\ell}}(-{1,1})$ *with* $\kappa ,{\ell}>0$*, where* ${H}^{\kappa }(-{1,1})$ *and* ${H}^{{\ell}}(-{1,1})$ *are Sobolev spaces on the domain* $\zeta ,\chi \in [-{1,1}]$. *Let* ${u}_{n,n}$* be the bivariate Legendre interpolant of* $u(\zeta ,\chi )$* at Legendre nodes on the interval* $[-{1,1}]$*, Then, the following error estimates hold:*

1. *Interpolation Error in the* ${L}_{2}$​*-Norm:*
  18 $${\parallel u-{u}_{n,n}\parallel }_{{L}_{2}(-1,1)\times (-1,1)}\le {C}_{1}{n}^{-min\left(\kappa ,{\ell}\right)}{\parallel u\parallel }_{{H}^{\kappa }\left(-{1,1}\right)\cap {H}^{{\ell}}\left(-{1,1}\right)},$$
2. *Error in Approximating the* $k$*-th Derivative:*
  19 $${\parallel {\partial }_{\zeta }^{k}u-{u}_{n,n}\parallel }_{{L}_{2}(-1,1)\times (-1,1)}\le {C}_{2}{n}^{-\kappa +k}{\parallel u\parallel }_{{H}^{\kappa }\left(-1,1\right)\cap {H}^{{\ell}}\left(-{1,1}\right)},$$
  *and*
  20 $$\parallel {\partial }_{\chi }^{k}u-{u}_{n,n}{\parallel }_{{L}_{2}\left(-1,1\right)\times \left(-1,1\right)}\le {C}_{3}{n}^{-{\ell}+k}\parallel u{\parallel }_{{H}^{\kappa }\left(-{1,1}\right)\cap {H}^{{\ell}}\left(-{1,1}\right)}.$$

## Mathematical formulation via Legendre collocation method

To solve the system (10)–(12) using egendre-collocation technique^35^, the solution domain should transformed from $[0,{L}_{\eta }]$ or $[0,{L}_{\xi }]$ to the interval $[-{1,1}]$. Hence, the linear transformations $\eta =\frac{1}{2}{L}_{\eta }\left(\zeta +1\right)$ and $\xi =\frac{1}{2}{L}_{\xi }\left(\chi +1\right)$, where $L\to \infty$ are applied. This yields the following coupled system:

21 $$\frac{{R_{1} }}{{R_{2} }}\left( {\frac{2}{{L_{\eta } }}} \right)f^{\prime\prime\prime} + \left( {\frac{{L_{\xi } \chi + L_{\xi } }}{{2 + L_{\xi } \chi + L_{\xi } }} + \frac{3}{4}} \right)ff^{\prime\prime} - \frac{1}{2}f^{\prime 2} + \frac{{R_{3} }}{{R_{2} }}\theta = \left( {\chi + 1} \right)\left( {f^{\prime}\frac{{\partial f^{\prime}}}{\partial \chi } - f^{\prime\prime}\frac{\partial f}{{\partial \chi }}} \right),$$

22 $$\frac{1}{\text{Pr}}\left(\frac{2}{{L}_{\eta }}\right)\left(\frac{{R}_{5}+{N}_{R}}{{R}_{4}}\right){\theta }^{\prime\prime} +\left(\frac{{L}_{\xi } \chi +{L}_{\xi }}{2+{L}_{\xi } \chi +{L}_{\xi }}+\frac{3}{4}\right)f{\theta }^{\prime}-\varepsilon {f}^{\prime}\theta =\left(\frac{2}{{L}_{\xi }}\right)\left(\chi +1\right)\left({f}^{\prime}\frac{\partial \theta }{\partial \chi }-{\theta }^{\prime}\frac{\partial f}{\partial \chi }\right),$$

with the boundary conditions

23 $$\begin{array}{c}f=0,{f'}=0,\theta =1 \; {\text{a}}{\text{t}} \; \zeta =-1,\\ {f'}=0,\theta =0 \; {\text{a}}{\text{t}} \; \zeta =1.\end{array}$$

To solve the system (21)–(23), it is necessary to use the following transformation:

24 $$g-{f}^{\prime}=0,$$

25 $$\frac{{R}_{1}}{{R}_{2}}\left(\frac{2}{{L}_{\eta }}\right){g}^{\prime\prime}+\left(\frac{{L}_{\xi } \chi +{L}_{\xi }}{2+{L}_{\xi } \chi +{L}_{\xi }}+\frac{3}{4}\right)f{g}^{\prime}-\frac{1}{2}{g}^{2}+\frac{{R}_{3}}{{R}_{2}}\theta =\left(\chi +1\right)\left(g\frac{\partial g}{\partial \chi }-{g}^{\prime}\frac{\partial f}{\partial \chi }\right),$$

26 $$\frac{1}{\text{Pr}}\left(\frac{2}{{L}_{\eta }}\right)\left(\frac{{R}_{5}+{N}_{R}}{{R}_{4}}\right){\theta }^{\prime\prime} +\left(\frac{{L}_{\xi } \chi +{L}_{\xi }}{2+{L}_{\xi } \chi +{L}_{\xi }}+\frac{3}{4}\right)f{\theta }^{\prime}-\varepsilon g\theta =\left(\frac{2}{{L}_{\xi }}\right)\left(\chi +1\right)\left(g\frac{\partial \theta }{\partial \chi }-{\theta }^{\prime}\frac{\partial f}{\partial \chi }\right),$$

with the boundary conditions

27 $$\begin{array}{c}f=0,{g}=0,\theta =1{\text{ at }}\zeta =-1,\\ g=0,\theta =0{\text{ at }}\zeta =1.\end{array}$$

Assume that the Legendre polynomials of the nonlinear system (21)–(23) provide the following approximate solution for the unknowns function:

$$f\left(\zeta ,\chi \right)\cong \sum_{l=0}^{n}\sum_{j=0}^{n}{c}_{l,j}{P}_{l}\left(\zeta \right){P}_{j}\left(\chi \right),$$

$$g\left(\zeta ,\chi \right)\cong \sum_{l=0}^{n}\sum_{j=0}^{n}{e}_{l,j}{P}_{l}\left(\zeta \right){P}_{j}\left(\chi \right),$$

28 $$\theta \left(\zeta ,\chi \right)\cong \sum_{l=0}^{n}\sum_{j=0}^{n}{d}_{l,j}{P}_{l}\left(\zeta \right){P}_{j}\left(\chi \right).$$

By substituting into the system and applying the collocation approach, results:

29 $$\sum_{l=0}^{n}\sum_{j=0}^{n}\left[{e}_{l,j}{P}_{l}({\zeta }_{i}){P}_{j}({\chi }_{k})-{c}_{l,j}{P}_{l}^{\prime}({\zeta }_{i}){P}_{j}({\chi }_{k})\right]=0,$$

$$\frac{{R}_{1}}{{R}_{2}}\left(\frac{2}{{L}_{\eta }}\right)\sum_{l=0}^{n}\sum_{j=0}^{n}{e}_{l,j} {P}_{l}^{{\prime}{\prime}}({\zeta }_{i}){P}_{j}({\chi }_{k})+\frac{{R}_{3}}{{R}_{2}}\sum_{l=0}^{n}\sum_{j=0}^{n}{d}_{l,j}{P}_{l}({\zeta }_{i}){P}_{j}({\chi }_{k})$$

$$+\sum_{l=0}^{n}\sum_{j=0}^{n}\sum_{\omega =0}^{n}\sum_{{\ell}=0}^{n}\left[\left(\frac{{L}_{\xi }{\chi }_{k}+{L}_{\xi }}{2+{L}_{\xi }{\chi }_{k}+{L}_{\xi }}+\frac{3}{4}\right){c}_{l,j}{e}_{\omega ,{\ell}} {P}_{\omega }^{\prime}\left({\zeta }_{i}\right)-\frac{1}{2}{e}_{l,j}{e}_{\omega ,{\ell}}{P}_{j}\left({\chi }_{k}\right)\right]{P}_{l}\left({\zeta }_{i}\right){P}_{\omega }\left({\zeta }_{i}\right){P}_{{\ell}}\left({\chi }_{k}\right)$$

30 $$=\left({\chi }_{k}+1\right)\left[\sum_{l=0}^{n}\sum_{j=0}^{n}\sum_{\omega =0}^{n}\sum_{{\ell}=0}^{n}\left({e}_{l,j}{e}_{\omega ,{\ell}}{P}_{l}\left({\zeta }_{i}\right)-{e}_{l,j}{c}_{\omega ,{\ell}}{P}_{l}^{\prime}\left({\zeta }_{i}\right)\right){P}_{j}\left({\chi }_{k}\right){P}_{\omega }\left({\zeta }_{i}\right){P}_{{\ell}}^{\prime}({\chi }_{k})\right],$$

$$\frac{1}{\text{Pr}}\left(\frac{2}{{L}_{\eta }}\right)\left(\frac{{R}_{5}+{N}_{R}}{{R}_{4}}\right)\sum_{l=0}^{n}\sum_{j=0}^{n}{d}_{l,j}{P}_{l}^{\prime\prime} ({\zeta }_{i}){P}_{j}({\chi }_{k})$$

$$+\sum_{l=0}^{n}\sum_{j=0}^{n}\sum_{\omega =0}^{n}\sum_{{\ell}=0}^{n}\left[\left(\frac{{L}_{\xi }{\chi }_{k}+{L}_{\xi }}{2+{L}_{\xi }{\chi }_{k}+{L}_{\xi }}+\frac{3}{4}\right){c}_{l,j}{d}_{\omega ,{\ell}}{P}_{\omega }^{\prime}({\zeta }_{i})-\varepsilon {e}_{l,j}{d}_{\omega ,{\ell}}{P}_{\omega }({\zeta }_{i})\right]{P}_{l}({\zeta }_{i}){P}_{j}({\chi }_{k}){P}_{{\ell}}({\chi }_{k})$$

31 $$=\left(\frac{2}{{L}_{\xi }}\right)\left({\chi }_{k}+1\right)\sum_{l=0}^{n}\sum_{j=0}^{n}\sum_{\omega =0}^{n}\sum_{{\ell}=0}^{n}\left[\begin{array}{c}{e}_{l,j}{d}_{k,{\ell}}{P}_{j}\left({\chi }_{k}\right){P}_{\omega }\left({\zeta }_{i}\right){P}_{{\ell}}^{\prime}\left({\chi }_{k}\right)\\ -{c}_{l,j}{d}_{\omega ,{\ell}}{P}_{j}^{\prime}\left({\chi }_{k}\right){P}_{\omega }^{\prime}\left({\zeta }_{i}\right){P}_{{\ell}}\left({\chi }_{k}\right)\end{array}\right]{P}_{l}\left({\zeta }_{i}\right),$$

with the boundary conditions

$$\sum_{l=0}^{n}\sum_{j=0}^{n}{c}_{l,j}{P}_{l}\left(-1\right){P}_{j}\left({\chi }_{k}\right)=0, \sum_{l=0}^{n}\sum_{j=0}^{n}{e}_{l,j}{P}_{l}\left(-1\right){P}_{j}\left({\chi }_{k}\right)=0, \sum_{l=0}^{n}\sum_{j=0}^{n}{d}_{l,j}{P}_{l}\left(-1\right){P}_{j}\left({\chi }_{k}\right)=0,$$

32 $$\mathop \sum \limits_{l = 0}^{n} \mathop \sum \limits_{j = 0}^{n} e_{l,j} \,P_{l} \left( 1 \right)P_{j} \left( {\chi_{k} } \right) = 0,\;\;{ }\mathop \sum \limits_{l = 0}^{n} \mathop \sum \limits_{j = 0}^{n} d_{l,j} \,P_{l} \left( 1 \right)P_{j} \left( {\chi_{k} } \right) = 0,$$

Where ${\chi }_{k}={\zeta }_{i}=\text{cos}\left(\left(\frac{n-i}{n}\right)\pi \right),i=0,1,2,...,n$. When combined the conditions (32) with the nonlinear system (30) and (31), the system matrix form is

33 $$\left[ {\begin{array}{*{20}c} {{\text{A}}_{11} } & {\text{O}} & {{\text{A}}_{13} } \\ {\text{O}} & {{\text{A}}_{22} } & {{\text{A}}_{23} } \\ {\text{O}} & {{\text{A}}_{32} } & {\text{O}} \\ \end{array} } \right]\left[ {\begin{array}{*{20}c} \text{C} \\ \text{D} \\ \text{E} \\ \end{array} } \right] + \left[ {\begin{array}{*{20}c} {\text{O}} & {\text{O}} & {\text{O}} \\ {\text{O}} & {\text{O}} & {\text{H}} \\ {\text{O}} & {\text{O}} & {\text{O}} \\ \end{array} } \right]\left[ {\begin{array}{*{20}c} \text{O} \\ \text{O} \\ {{\overline{\text{E}}}} \\ \end{array} } \right]\, + \left[ {\begin{array}{*{20}c} {\text{O}} & {\text{O}} & {\text{O}} \\ {\text{O}} & {{\text{B}}_{22} } & {\text{O}} \\ {{\text{B}}_{31} } & {\text{O}} & {{\text{B}}_{32} } \\ \end{array} } \right]\left[ {\begin{array}{*{20}c} {\rm K} \\ \Psi \\ {\rm T} \\ \end{array} } \right] = \left[ {\begin{array}{*{20}c} \text{O} \\ \text{O} \\ \text{F} \\ \end{array} } \right],$$

$${\text{A}}_{11}={\left[\begin{array}{cccccccc}{\delta }_{1,0}& {\delta }_{1,1}& \dots & {\delta }_{n,n}& {P}_{l}(-1){P}_{j}({\chi }_{0})& {P}_{l}(-1){P}_{j}({\chi }_{1})& \dots & {P}_{l}(-1){P}_{j}({\chi }_{n})\end{array}\right]}^{t},$$

$${\text{A}}_{13}={\left[\begin{array}{cccc}{\alpha }_{1,0}& {\alpha }_{1,1}& \begin{array}{cc}\dots & {\alpha }_{n,n}\end{array}& \text{O}\end{array}\right]}^{t},{\alpha }_{i,k}={P}_{l}\left({\zeta }_{i}\right){P}_{j}\left({\chi }_{k}\right),{\delta }_{i,k}=-{P}_{l}^{\prime}\left({\zeta }_{i}\right){P}_{j}\left({\chi }_{k}\right),$$

$${\text{A}}_{22}={\left[\begin{array}{cccc}{\mu }_{1,0}& {\mu }_{1,1}& \begin{array}{cc}\dots & {\mu }_{n-1,n}\end{array}& \begin{array}{cccccc}{P}_{l}(1){P}_{j}({\chi }_{0})& \dots & {P}_{l}(1){P}_{j}({\chi }_{n})& {P}_{l}(-1){P}_{j}({\chi }_{0})& \dots & {P}_{l}(-1){P}_{j}({\chi }_{n})\end{array}\end{array}\right]}^{t},$$

$${\text{A}}_{23}=\left[\begin{array}{cccc}{\kappa }_{1,0}& {\kappa }_{1,1}& \begin{array}{cc}\dots & {\kappa }_{n-1,n}\end{array}& \text{O}\end{array}\right],{\kappa }_{i,k}=\frac{{R}_{1}}{{R}_{2}}\left(\frac{2}{{L}_{\eta }}\right){P}_{l}^{\prime\prime} \left({\zeta }_{i}\right){P}_{j}\left({\chi }_{k}\right),$$

$${\text{A}}_{32}={\left[\begin{array}{cccc}{\gamma }_{1,0}& {\gamma }_{1,1}& \begin{array}{cc}\dots & {\gamma }_{n-1,n}\end{array}& \begin{array}{cccccc}{P}_{l}(1){P}_{j}({\chi }_{0})& \dots & {P}_{l}(1){P}_{j}({\chi }_{n})& {P}_{l}(-1){P}_{j}({\chi }_{0})& \dots & {P}_{l}(-1){P}_{j}({\chi }_{n})\end{array}\end{array}\right]}^{t},$$

$$\text{F}={\left[\begin{array}{ccc}\text{O}& 1\,\,\,1\cdots 1& \text{O}\end{array}\right]}^{t}, {\gamma }_{i,k}=\frac{1}{\text{Pr}}\left(\frac{2}{{L}_{\eta }}\right)\left(\frac{{R}_{5}+{N}_{R}}{{R}_{4}}\right){P}_{l}^{\prime\prime}\left({\zeta }_{i}\right){P}_{j}\left({\chi }_{k}\right),$$

$$\text{H}={\left[\begin{array}{cccc}{h}_{1,0}& {h}_{1,1}& \begin{array}{cc}\dots & {h}_{n-1,n}\end{array}& \text{O}\,\,\,\,\text{O}\end{array}\right]}^{t},{h}_{i,k}={P}_{l}\left({\zeta }_{i}\right){P}_{j}\left({\chi }_{k}\right){P}_{\omega }\left({\zeta }_{i}\right)\left(-\frac{1}{2}{P}_{{\ell}}\left({\chi }_{k}\right)-\left({\chi }_{k}+1\right){P}_{{\ell}}^{\prime}\left({\chi }_{k}\right)\right),$$

$$B_{22} = \left[ {\begin{array}{*{20}c} {\hbar_{1,\,0} } & {\hbar_{1,\,1} } & {\begin{array}{*{20}c} \ldots & {\hbar_{n - 1,\,n} } \\ \end{array} } & {\text{O}\,\,\,\text{O}} \\ \end{array} } \right]^{t} , B_{31} = \left[ {\begin{array}{*{20}c} { \omega_{1,\,0} } & {\omega _{1,\,1} } & {\begin{array}{*{20}c} \ldots & { \omega_{n - 1,\,n} } \\ \end{array} } & {\text{O}\,\,\,\text{O}} \\ \end{array} } \right]^{t} ,$$

$${\hslash }_{i,k}={P}_{j}\left({\chi }_{k}\right)\left[\left(\frac{{L}_{\xi }{\chi }_{k}+{L}_{\xi }}{2+{L}_{\xi }{\chi }_{k}+{L}_{\xi }}+\frac{3}{4}\right){P}_{l}\left({\zeta }_{i}\right){P}_{\omega }{\prime}\left({\zeta }_{i}\right){P}_{{\ell}}\left({\chi }_{k}\right)+\left({\chi }_{k}+1\right){P}_{l}^{\prime}\left({\zeta }_{i}\right){P}_{\omega }\left({\zeta }_{i}\right){P}_{{\ell}}^{\prime}\left({\chi }_{k}\right)\right],$$

$$\omega_{i,k} = P_{l} \left( {\zeta_{i} } \right)P_{\ell } \left( {\chi_{k} } \right)\left[ {\left( {\frac{{L_{\xi } \chi_{k} + L_{\xi } }}{{2 + L_{\xi } \chi_{k} + L_{\xi } }} + \frac{3}{4}} \right)\,P_{j} \left( {\chi_{k} } \right)P_{\omega }^{\prime} \,\left( {\zeta_{i} } \right)\, + \left( {\frac{2}{{L_{\xi } }}} \right)\left( {\chi_{k} + 1} \right)P_{j}^{\prime} \left( {\chi_{k} } \right)P_{\omega }^{\prime} \left( {\zeta_{i} } \right)} \right],$$

$${\text{B}}_{33}={\left[\begin{array}{cccc}{\lambda }_{1,0}& {\lambda }_{1,1}& \begin{array}{cc}\dots & {\lambda }_{n-1,n}\end{array}& \text{O}\,\,\,\text{O}\end{array}\right]}^{t},$$

$${\lambda }_{i,k}={P}_{l}\left({\zeta }_{i}\right){P}_{j}\left({\chi }_{k}\right){P}_{\omega }\left({\zeta }_{i}\right)\left[-\varepsilon {P}_{{\ell}}\left({\chi }_{k}\right)-\left(\frac{2}{{L}_{\xi }}\right)\left({\chi }_{k}+1\right){P}_{{\ell}}^{\prime}\left({\chi }_{k}\right)\right],$$

$$\text{C}={\left[\begin{array}{cccc}{c}_{\text{0,0}}& {c}_{\text{1,0}}& \dots & {c}_{n,n}\end{array}\right]}^{t},\text{D}={\left[\begin{array}{cccc}{d}_{\text{0,0}}& {d}_{1,0}& \dots & {d}_{n,n}\end{array}\right]}^{t},\text{E}={\left[\begin{array}{cccc}{e}_{\text{0,0}}& {e}_{\text{1,0}}& \dots & {e}_{n,n}\end{array}\right]}^{t},$$

$$\overline{\text{E} }={\left[\begin{array}{cccc}{e}_{\text{0,0}}^{2}& {e}_{\text{1,0}}{e}_{\text{0,0}}& \dots & {e}_{n,n}^{2}\end{array}\right]}^{t},\text{ K}={\left[\begin{array}{cccc}{c}_{\text{0,0}}{d}_{\text{0,0}}& {c}_{\text{1,0}}{d}_{\text{0,0}}& \dots & {c}_{n,n}{d}_{n,n}\end{array}\right]}^{t},$$

$$\Psi ={\left[\begin{array}{cccc}{c}_{\text{0,0}}{e}_{\text{0,0}}& {c}_{\text{1,0}}{e}_{\text{0,0}}& \dots & {c}_{n,n}{e}_{n,n}\end{array}\right]}^{t},\text{T}={\left[\begin{array}{cccc}{e}_{\text{0,0}}{d}_{\text{0,0}}& {e}_{\text{1,0}}{d}_{\text{0,0}}& \dots & {e}_{n,n}{d}_{n,n}\end{array}\right]}^{t}.$$

In the nonlinear system (33), C, D and E are the unknowns. We’ll apply Newton’s method to solve resulting nonlinear system that has $3n+3$ equations with a tolerance of ${10}^{-15}$. Convergence was verified by increasing the number of collocation points $n$ until the residual error fell below ${10}^{-10}$. Once the system has been resolved, the values of the unknowns are known. To solve our problem, the inverse transformations.

34 $$\zeta =\frac{2}{{L}_{\eta }}\eta -1 \; \text{and} \; \chi =\frac{2}{{L}_{\xi }}\xi -1,$$

should be applied to obtain the tables and the graphs to introduce the physical and mechanical interpretations. All computations were performed in Mathematica 13.3 using a custom spectral collocation code, with derivatives computed via Legendre polynomial differentiation matrices.

Tables 3 and 4 demonstrate the validation of the numerical method used in this study and compare the results with other research in^14,15,36–41^ with $\varepsilon =\phi ={N}_{R}=0$. Here, $\xi ={10}^{4}$ is indicated by $\infty$.

**Table 3:** Comparison of ${f}^{\prime\prime} (0,\text{0)}$ and $-{\theta }^{\prime}(0,0)$ at $\varepsilon =\phi ={N}_{R}=0$ for different Prandtl number values (0.1, 1.0, 10).

$f^{{\prime}{\prime}}(\text{0,0})$			$-\theta^ {\prime}(\text{0,0})$					
$\text{Pr}$	Cebeci and Bradshaw^36^	Present results	Na^37^	Na and Chiou^38^	Cebeci and Bradshaw^36^	Kays and Crawford^39^	Lin and Chen^40^	Present results
0.1	1.2104	$1.2150$	–	–	0.1637	0.164	0.1627	0.162733
1.0	0.9081	0.9081	0.4010	0.4011	0.4009	0.4010	0.4009	0.401011
10	0.5930	0.5926	0.8269	0.8269	0.8266	0.8270	0.8258	0.826651

**Table 4:** Comparison of ${f}^{\prime\prime} (\infty , {0}\text{)}$ and $-\theta (\infty ,0)$ at $\varepsilon =\phi ={N}_{R}=0$ for different Prandtl number values.

$f^{{\prime}{\prime}}(0,\infty )$				$-\theta ^{\prime}(0,\infty )$					
$\text{Pr}$	Hering and Grosh^15^	Roy^14^	Present results	Na^37^	Hering and Grosh^15^	Roy^14^	Alamgir^41^	Na and Chiou^38^	Present results
0.1	1.0960	–	1.0958	0.2113	–	0.2141	–	0.2113	0.21132
1.0	0.7694	0.8600	0.7694	0.5104	0.5275	0.5280	0.5104	0.5104	0.51045
10	–	0.4899	0.4876	–	1.0354	1.0159	1.0340	–	1.03397

## Results and discussion

**Fig. 3:**
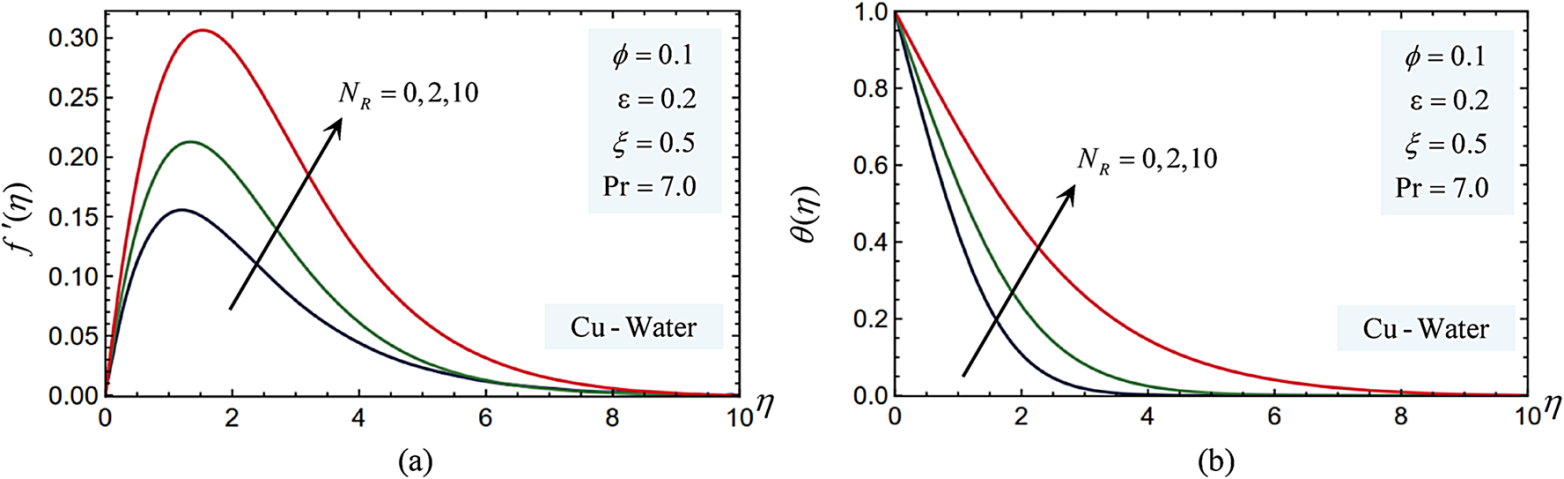
(**a**) The radiation parameter impacts on the velocity profiles ${f}^{\prime}(\eta )$. (**b**) The radiation parameter impacts on the temperature $\theta (\eta )$ profiles.

**Fig. 4:**
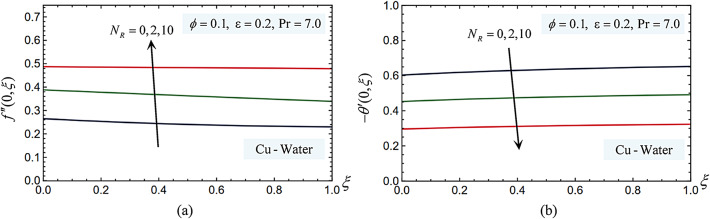
(**a**) The radiation parameter affects the skin-friction coefficient. (**b**) The radiation parameter effects on the Nusselt number.

**Fig. 5:**
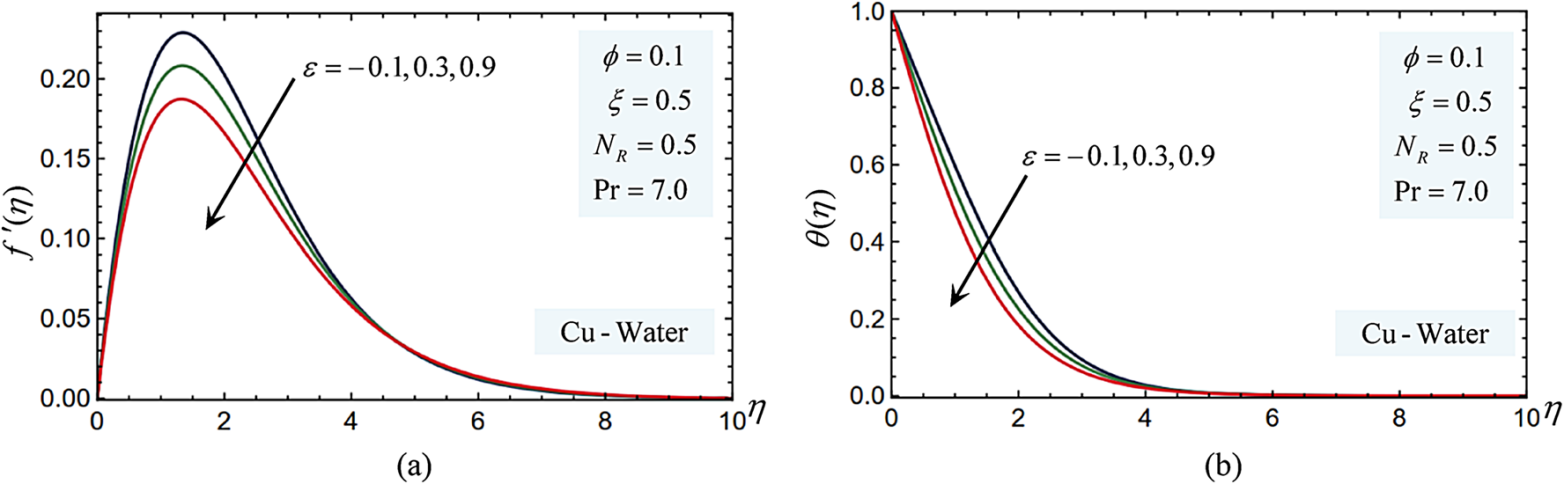
(**a**) The pressure work parameter impacts on the velocity profiles ${f}^{\prime}\left(\eta \right)$. (**b**) The pressure work parameter impacts on the temperature $\theta (\eta )$ profiles.

**Fig. 6:**
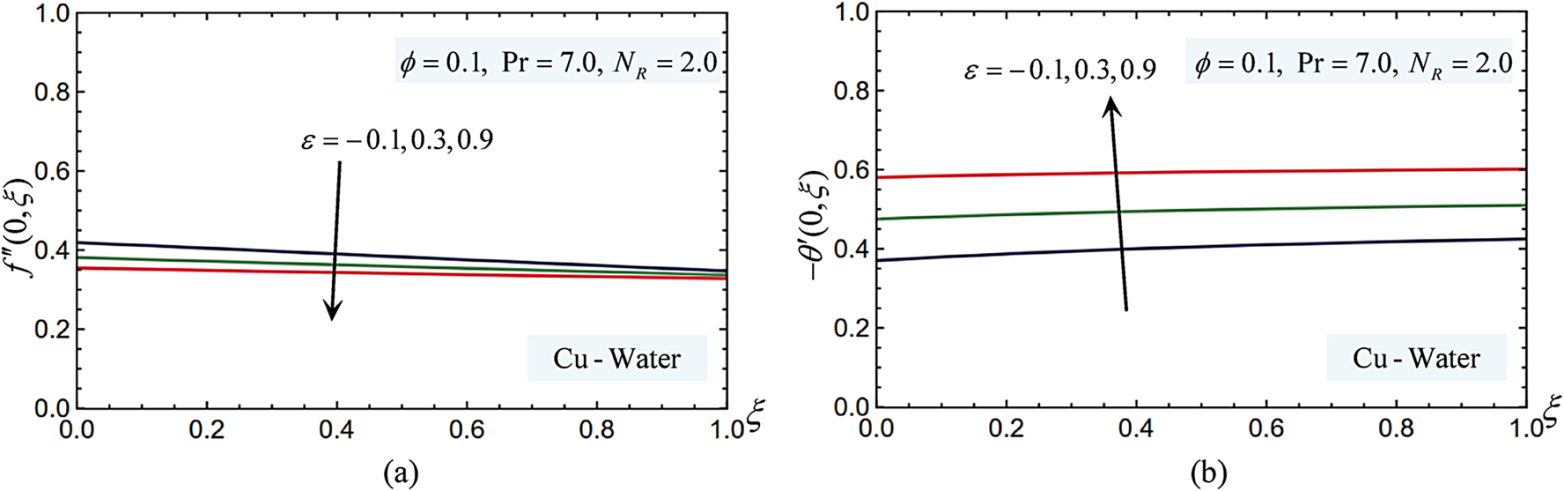
(**a**) The pressure work parameter affects the skin-friction coefficient. (**b**) The pressure work parameter effects on the Nusselt number.

**Fig. 7:**
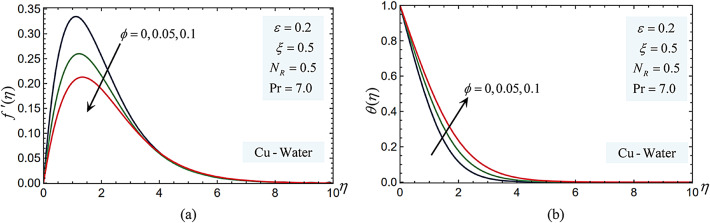
(**a**) The nanoparticle volume fraction parameter impacts on the velocity profiles $f^{\prime}(\eta )$. (**b**) The nanoparticle volume fraction impacts on the temperature $\theta (\eta )$ profiles.

**Fig. 8:**
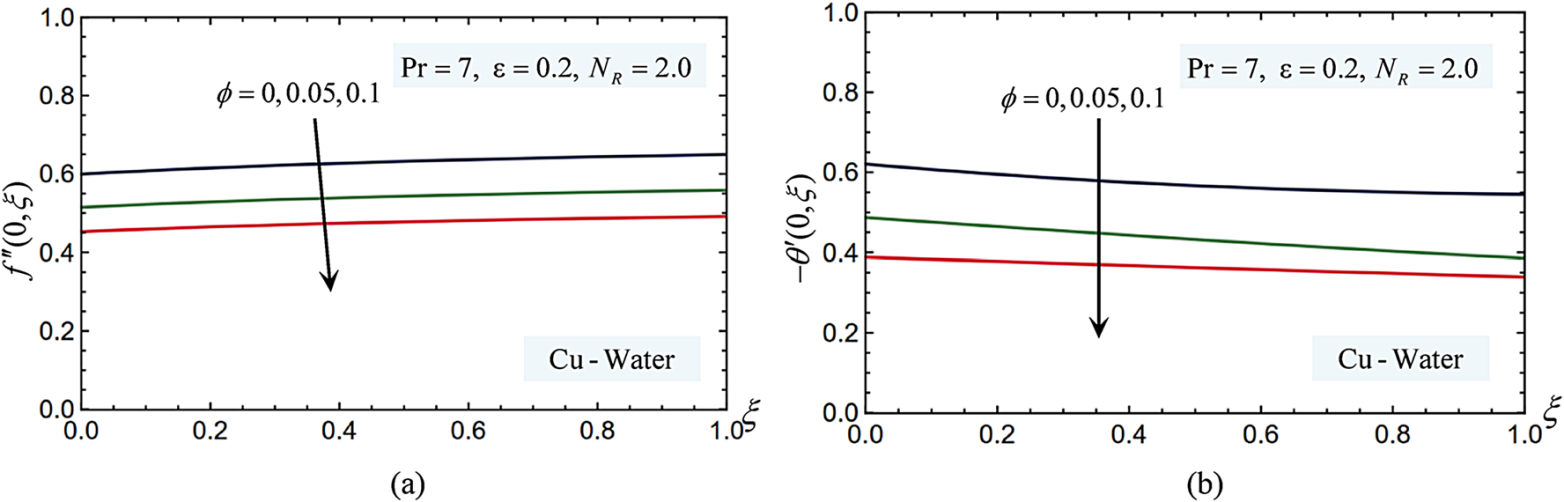
(**a**) The nanoparticle volume fraction parameter affects the skin-friction coefficient. (**b**) The nanoparticle volume fraction parameter affects the Nusselt number.

**Fig. 9:**
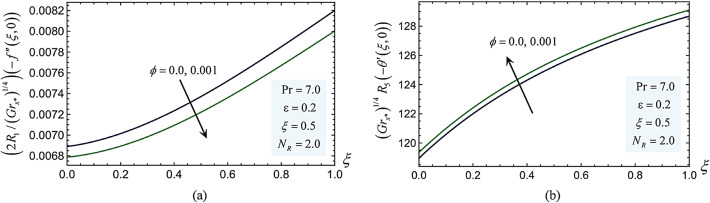
(**a**) The skin-friction values at $\phi =0$ and $\phi =0.001$. (**b**) The Nusselt values at $\phi =0$ and $\phi =0.001$.

**Fig. 10:**
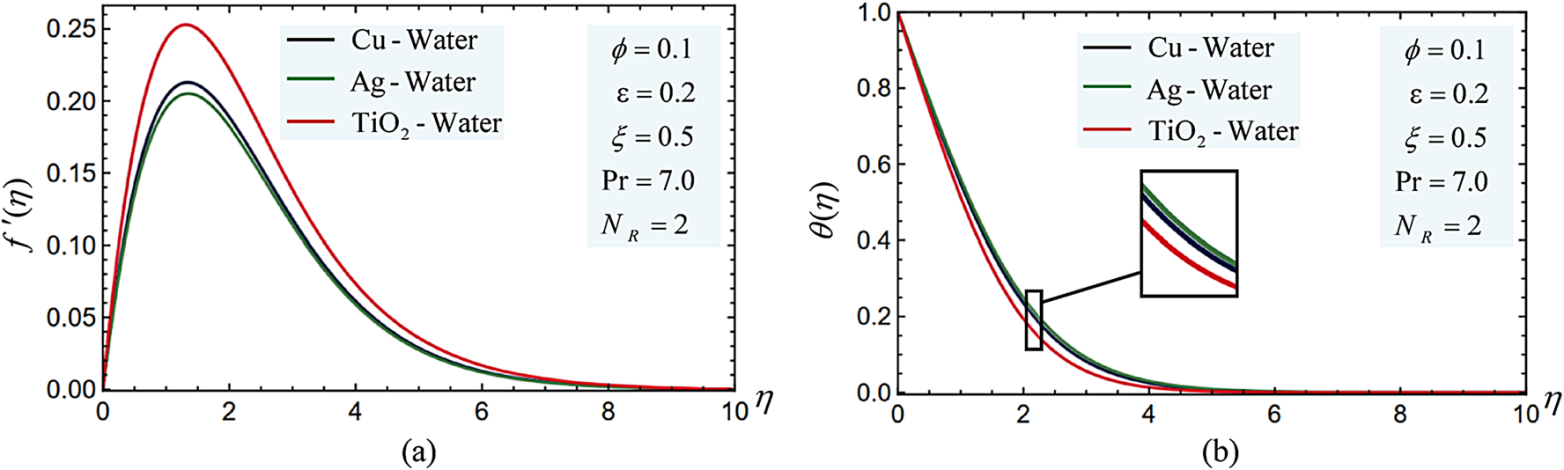
(**a**) The different nanoparticle type impacts on the velocity profiles $f^{\prime}(\eta )$. (**b**) The different nanoparticle type impacts on the temperature $\theta (\eta )$ profiles.

**Fig. 11:**
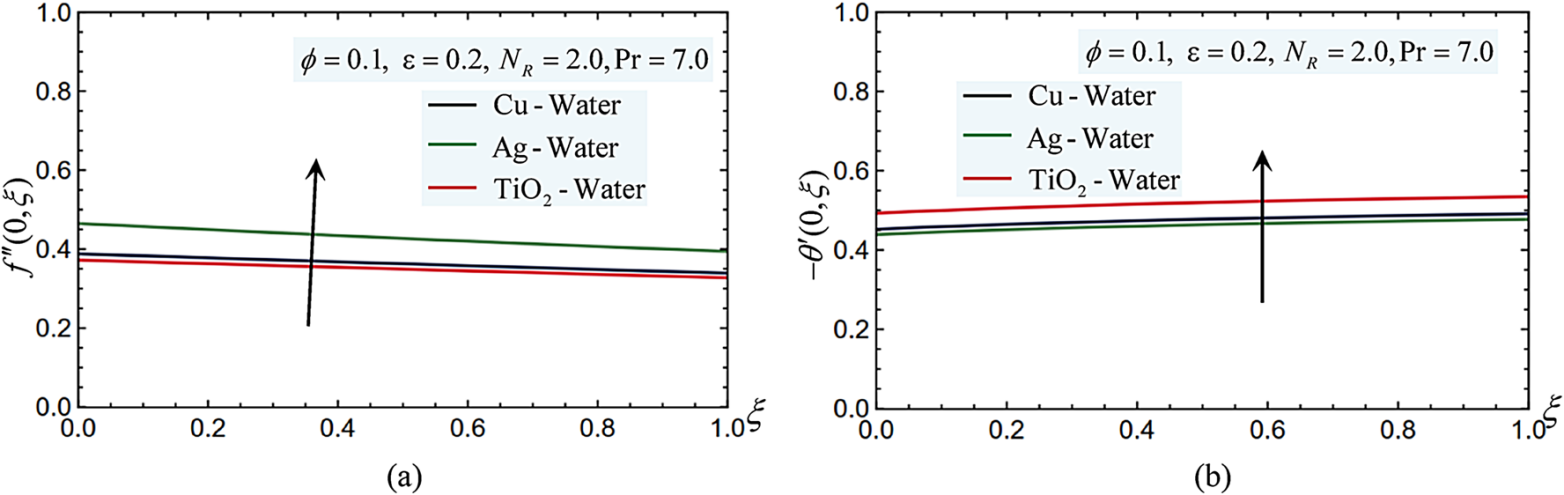
(**a**) The different nanoparticle type affects the skin-friction coefficient. (**b**) The different nanoparticle type effects on the Nusselt number.

The Eqs. (10) and (11), which satisfy the boundary conditions in Eq. (12), have been numerically solved using the Legendre collocation method for a range of values of the involved parameters, including the Prandtl number, pressure work parameter, radiation parameter, and nanoparticle volume fraction. Figures 3, 4, 5, 6, 7, 8, 9, 10 and 11 display the effects of varying the radiation parameter, pressure work parameter, nanoparticle volume percentage, and nanoparticle type on the local skin friction, Nusselt number dimensionless temperature, $\theta (\eta )$, and dimensionless velocity profiles, ${f'}(\eta )$. Three distinct kinds of nanoparticles nanoparticles—Cu, Ag, and TiO_2_—were examined, using water as the base fluid. The components Cu, Ag, and TiO_2_ as well as the thermophysical properties of water are displayed in Table 1. The effects of the parameters used in this study are:

### Radiation parameter

Figure 3 illustrates how changing the radiation parameter ${N}_{R}$ effects on the Cu-water nanofluid’s dimensionless temperature and velocity within the boundary layer in relation to $\eta$. It has been noted that the temperature and velocity inside the boundary layer rise with increasing ${N}_{R}$. Physically, a higher ${N}_{R}$ causes the nanofluid to absorb more heat from radiation, which raises the fluid’s temperature and causes it to flow more quickly because of its decreased viscosity. This may result in improved heat transmission and modifications to the system’s fluid dynamics.

Figure 4 introduces how the radiation parameter affects the local skin friction coefficient and local Nusselt number for Cu nanoparticles. According to these statistics, a higher radiation parameter value increases the velocity gradient but reduces buoyancy effects by enhancing energy dissipation via radiation, leading to a decrease in the skin friction coefficient. Furthermore, increasing leads to a decrease in local Nusselt number because a portion of the heat is carried away by radiation, reducing the convective heat transfer, which indicates that the surface’s strength and hardness will decrease when thermal radiation is present.

### Pressure work parameter

The temperature and velocity profiles for the pressure work parameter in the Cu-water nanofluid scenario are displayed in Fig. 5. It is evident from these data that when $\varepsilon$ increases, the temperature and velocity profiles decline. Physically, the nanofluid becomes compressed when pressure rises, which lowers the fluid’s capacity to retain heat and causes the temperature to drop. The fluid is facing more flow resistance when the pressure gradient rises. The velocity is slowed down because the nanofluid must resist more forces to continue moving. Greater pressure gradients tend to decrease the fluid’s momentum, which means that for the fluid molecules to move, they must resist these greater pressure forces. Consequently, the boundary layer’s velocity profile falls.

Figure 6 shows how the pressure work parameter affects the local Nusselt number and skin friction coefficient. These numbers demonstrate that a higher-pressure work parameter results in lower skin friction coefficient values because higher-pressure work parameter can lead to a reduction in fluid viscosity (for temperature-dependent viscosity models), which may result in a lower wall shear stress and hence a lower skin friction coefficient. Furthermore, a higher-pressure work parameter results in higher local Nusselt numbers, indicating that the surface’s strength and hardness will be satisfactory when this force is present.

Table 5 presents how the skin friction coefficient and Nusselt number are affected by the pressure work parameter for both truncated and full cones. In both the truncated and complete cones, the skin friction reduces and the Nusselt number increases because of raising the pressure work parameter. Compared to the full cone, the truncated cone displays a reduced rate of heat transmission due to thicker boundary layer and reduced temperature gradient. Although the skin friction coefficients of the two are almost equal, the truncated cone’s coefficient is still lower than the full cone’s coefficient due to less steep velocity gradient and delayed boundary layer.

**Table 5:** Comparison of skin friction and Nusselt number for both truncated and full cone at $\text{Pr}=7,\phi =0.1,{N}_{R}=2$ for different pressure work parameters.

$\varepsilon$	Truncated Cone		Full Cone	
	${C}_{f}$	$N{u}_{{x}^{*}}$	${C}_{f}$	$N{u}_{{x}^{*}}$
$-0.1$	0.006754	110.9965	$0.007687$	$128.0992$
$0.3$	0.006731	126.6357	$0.007421$	$143.2174$
$0.9$	0.006704	145.7653	$0.007098$	$161.6534$

### Nanoparticle volume

Figure 7 illustrates how changing the volume percentage of nanoparticles affects the dimensionless temperature and velocity for the Cu-water nanofluid versus $\eta$. It is noted that when the value of $\phi$ grows, the temperature rises, and the velocity profiles fall. As the $\phi$ value rises, the fluid becomes more viscous. Natural convection is reduced as a result, causing the fluid to travel more slowly. The thermal boundary layer thickens because of this velocity decrease, raising the boundary layer’s internal temperature. Nanoparticles improve the effective thermal conductivity of the fluid, leading to higher temperature gradients near the heated surface and thinner thermal boundary layers due to improved heat diffusion. Generally, more nanoparticles enhance convective heat transfer and raise the temperature in the fluid.

Figure 8 demonstrates how, in the instance of Cu nanoparticles, rising $\phi$ from 5 to 10% causes the values of the temperature and velocity gradients at the surface to gradually diminish. However, when the concentration of nanoparticles in the base fluid increases, both heat transmission and skin friction increase. Therefore, it can be concluded that a nanofluid containing 10% nanoparticles has a greater impact on mechanical characteristics than one containing 5% nanoparticles. Using a nanofluid throughout the cooling process can generally improve the surface’s mechanical properties more actively. For instance, compared to pure water, the heat transfer rate is increased by 10–40% when a nanofluid is used. This accelerates the process of cooling and increases the strength and hardness of the surface.

As the volume fraction $\phi$ increases, the effective viscosity ${\mu }_{nf}$ rises according to the Brinkman model ${\mu }_{nf}/{\mu }_{f} ={\left(1-\phi \right)}^{-2.5}$. The nanofluid exhibits Newtonian fluid behavior at low $\phi$ (0.05–0.10), which increases heat transfer through improved thermal conductivity ${k}_{nf}$ and a slight rise in viscosity. Higher $\phi$, on the other hand, might cause non-Newtonian, slurry-like behavior due to nanoparticle aggregation and greater particle–particle interactions. This behavior is typified by exponentially increasing viscosity, possible sedimentation, and decreased convective flow. The size, shape, density, and dispersion stability of the nanoparticles determine the threshold concentration for the slurry transition. The highest packing fraction ${\phi }_{\text{max}}$ for stable solutions of spherical nanoparticles (Cu, Ag, TiO_2_) is often between 0.20 and 0.30. Beyond this, the nanofluid has a high flow resistance and resembles a slurry. Sedimentation dangers rise with increasing phi, especially for Ag and Cu, due to the differential in densities. Due to their high densities and aggregation tendencies, Cu-water and Ag-water nanofluids may exhibit slurry-like behavior at $\phi = 0.15{-}0.18$, but TiO_2_-water, which has a lower density, may stay stable until $\phi \approx 0.20{-}0.22$. In practical applications, such as cooling systems or heat exchangers, a slurry-like nanofluid ($\phi$ > 0.20) would complicate the use of pressure work-driven convection and negate thermal gains by increasing pumping power needs and pressure losses. The skin friction and Nusselt values for $\phi = 0$ and $\phi = 0.001$ are introduced in Fig. 9. As was said in the preceding explanation, it shows how increasing viscosity by raising $\phi$ causes the skin friction values to decrease. Conversely, an improvement in the heat transfer rate by increasing $\phi$ causes the Nusselt values to rise.

### Nanoparticles type

The fluid temperature and velocity profiles for Cu-, Ag-, and TiO_2_-water nanofluids are displayed in Fig. 10. Because the mass density of Cu is smaller than Ag but more than TiO_2_, it is found that the addition of TiO_2_ nanoparticles causes the fluid to move faster, whereas Ag nanoparticles cause the fluid to move more slowly than other nanoparticles before decreasing the velocity to zero. However, as seen in Table 1, the high value of Ag’s thermal conductivity raises the fluid temperature while TiO_2_ nanoparticles cause it to drop.

Figure 11 makes it evident that when the nanoparticle was changed from TiO_2_, Cu to Ag, the velocity gradient values at the surface grew progressively. However, the reverse order of the nanoparticle type happens with the temperature gradient. Then, Ag-nanofluid has a higher skin friction and surface shear stress than Cu and TiO_2_-nanofluid, respectively. It also Ag -nanofluid has a higher Nusselt number and a higher rate of heat transfer from the surface than Cu and TiO_2_-nanofluid, respectively. These findings suggest that Ag-nanofluid is a more effective cooling medium for surface hardness and strength.

## Conclusions

This study uses three different types of nanoparticles (Cu, Ag, and TiO_2_) to investigate the constant, laminar-free convection of a nanofluid from a truncated cone in the presence of heat radiation and pressure work. Our study’s objective was to determine the surface’s mechanical qualities and heat transfer characteristics, and the following findings were attained:

1. As the radiation parameter increases and the pressure work parameter, Prandtl number, and concentration of nanoparticles drop, the velocity within the boundary layer increases.
2. As the radiation parameter and nanoparticle concentration rise and the pressure work parameter and Prandtl number fall, the temperature inside the boundary layer rises.
3. By adding nanoparticles, the fluid velocity slows down. Yet, compared to other nanoparticles, TiO_2_ nanoparticles allow for quicker nanofluid mobility. Conversely, the high value of Ag’s thermal conductivity (Table 1) raises the fluid’s temperature, whereas TiO_2_ nanoparticles cause it to drop.
4. Using nanofluid as a cooling medium can increase mechanical characteristics (hardness and strength) by 10–40%, depending on the type and nanoparticles concentration used.
5. We limited our analysis to three types of nanoparticles, and the findings indicated that the best kind for improving the mechanical properties of the surface (increasing the heat flux) was Ag-nanofluid, while the best type for decreasing the surface shear stress was Ag-nanofluid.
6. The mechanical characteristics of the cone surface are adversely affected when heat radiation is present during the cooling process. Conversely, the mechanical characteristics of the surface are positively impacted by the presence of pressure work.
7. Selecting a coolant with a higher $\text{Pr}$ number helps enhance the cooling of hot surfaces.

## Electronic supplementary material

Below is the link to the electronic supplementary material.

Supplementary Material 1

## Data Availability

No publicly available repositories or databases are suitable for the current data submission. All data supporting the results of this study are available in the article. They can also be obtained from the corresponding author, MF, upon reasonable request.

## List of symbols

${C}_{p}$: Specific heat at constant pressure $(\frac{J}{\text{kg} \text{K}})$
${C}_{fx}$: Local skin friction (–)
$f$: Dimensionless stream function (–)
${\text{g}}$: Acceleration due gravity $(\text{m}/{\text{s}}^{2})$
$G{r}_{{x}^{*}}$: The local Grashof number (–)
$$k$$: Thermal conductivity $$(\text{W}/\text{mK})$$
$${N}_{R}$$: Radiation parameter (–)
$N{u}_{{x}^{*}}$: The local Nusselt number coefficient (–)
$$\text{Pr}$$: Prandtl number (–)
$$p$$: Fluid pressure $$(\text{N}/{\text{m}}^{2})$$
$r$: Local radius of the truncated cone $(\text{m})$
${x}_{0}$: Distance of the leading edge of truncated cone measured from the origin $(\text{m})$
${R}_{1}$: Ratio between the viscosities of the nanofluid and the base fluid (–)
${R}_{2}$: Ratio between the densities of the nanofluid and the base fluid (–)
${R}_{3}$: Ratio between $(\rho \beta )$ of the nanofluid and $(\rho \beta )$ of the base fluid (–)
${R}_{4}$: Ratio between the heat capacities of the nanofluid and the base fluid (–)
${R}_{5}$: Ratio between the thermal conductivities of the nanofluid and the base fluid (–)
$T$: Temperature of the fluid $(\text{K})$
${T}_{\infty }$: Temperature of the ambient fluid $(\text{K})$
$u$: Velocity component in the $x$-direction $(\text{m}/\text{s})$
$\nu$: Velocity component in the $y$-direction $(\text{m}/\text{s})$
${q}_{r}$: Radiation heat flux $(\text{W}/{\text{m}}^{2})$
$x$: Distance measured from the leading edge $(\text{m})$
$y$: Normally distance to the surface $(\text{m})$

## Greek symbols

$\alpha$: The thermal diffusivity $({\text{m}}^{2}/\text{s})$
${\alpha }^{*}$: Mean absorption coefficient $(1/ \text{m})$
$\beta$: Coefficient of thermal expansion $(1/\text{K})$
$\gamma$: The cone apex half-angle $(\text{rad})$
$\varphi$: Nanoparticles volume fraction (–)
$\eta$: The pseudo- similarity variable (–)
$$\xi$$: Dimensionless distance (–)
$\sigma$: Stefan Boltzmann constant $(\frac{{\text{W}}}{{{\text{m}}^{2} {\text{K}}^{ - 4} }})$
$$\upsilon$$: Kinematic viscosity $$({\text{m}}^{2}/\text{s})$$
$$\mu$$: Dynamic viscosity $$(\text{Pa} \text{s})$$
$$\theta$$: Dimensionless temperature (–)
$$\rho$$: Mass density $$(\text{kg}/{\text{m}}^{3})$$
$$\psi$$: Stream function $$({\text{m}}^{2}/\text{s})$$
$\varepsilon$: The pressure work parameter $(\text{kg } { \text{m}}^{2}/{\text{s}}^{2})$

## Subscripts

$f$: Base fluid conditions
$$nf$$: Nanofluid conditions
$$s$$: Nanoparticles conditions
$$w$$: Wall conditions
$$\infty$$: Ambient condition
