# Supplementary material for: Thermal radiation effects on nanofluid flow over a vertical cone in the presence of pressure work
Source: Sci Rep. 2025 Aug 4;15:28390. doi: 10.1038/s41598-025-10554-5 (PMC12322182; doi:10.1038/s41598-025-10554-5)
Supplement: Supplementary file 1 — Supplementary Material 1 [file 41598_2025_10554_MOESM1_ESM.docx]

**Appendix A: Detailed Derivation of the Pressure Work Term**

In the laminar free convection flow of a nanofluid around a truncated cone, the energy contribution from pressure forces due to thermal expansion is considered by the pressure work component $\beta_{nf}Tu\frac{\partial p}{\partial x}$ in equation (3). To explain this term's presence in the energy equation, a thorough derivation is given in this appendix.

1. **The setting of General Energy Equation**

Convection, conduction, and source factors like pressure work and radiative heat flow are all included in the fluid's energy equation, which explains the equilibrium of thermal energy. The energy equation for a nanofluid in free convection, ignoring viscous dissipation (as is common in boundary layer flows), is as follows:

| $\left( \rho C_{p} \right)_{nf}\frac{DT}{Dt} =k_{nf}\nabla^{2}T+source/sink term,$ | (A1) |
| --- | --- |

where $DT/Dt$ is the material derivative of pressure and where $\left( \rho C_{p} \right)_{nf}$ is the heat capacity of the nanofluid, $T$ is the temperature, and $k_{nf}$ is the thermal conductivity of the nanofluid. Pressure work and radiative heat flow are examples of source/sink terminology. Equation (A1) can be written as

| $\left( \rho C_{p} \right)_{nf}\left( u\frac{\partial T}{\partial x}+v\frac{\partial T}{\partial y} \right)=k_{nf}\frac{\partial^{2}T}{\partial y^{2}}+Pressure work -\frac{\partial q_{r}}{\partial y},$ | (A2) |
| --- | --- |

where $u$ and $v$ are velocity components in the $x$ and $y$-directions and $\partial q_{r}/\partial y$ is the  thermal radiation effects term.

1. **Pressure Work in Fluid Dynamics**

When a fluid element experiences volume changes, especially because of thermal expansion in free convection, pressure work is the mechanical work performed by pressure forces. The dot product of the velocity vector $\mathbf{u} = (u, v)$ and the pressure gradient $\nabla p$ yields the work performed per unit volume by pressure forces:

| $Pressure work=\mathbf{u}\cdot\nabla p=u\frac{\partial p}{\partial x}+v\frac{\partial p}{\partial y} .$ | (A3) |
| --- | --- |

The pressure gradient in the $y$-direction (normal to the surface) is usually insignificant $\partial p/\partial y\approx0$ in the boundary layer approximation for free convection over a vertical surface or cone because the vertical direction aligned with gravity dominates the pressure variation. The pressure work thus reduces to $u \partial p/\partial x.$

1. **Incorporation of Thermal Expansion**

The coefficient of volume expansion $\beta$ measures the temperature differences that cause volume changes in free convection. When $T$ is the local temperature, the work related to thermal expansion is proportional to $\beta T$. The effective coefficient of volume expansion for a nanofluid, which takes into consideration the presence of nanoparticles, is $\beta_{nf}$. The pressure work phrase is therefore changed to $\beta_{nf}Tu \partial p/\partial x$. The energy contribution from pressure forces acting on a thermally expanding fluid element traveling in the $x$-direction is represented by this quantity.

1. **Determination of the Pressure Gradient**

In free convection, the hydrostatic balance in the ambient fluid outside the boundary layer determines the pressure gradient in the $x$-direction (aligned with the cone surface and gravity). Assuming the ambient fluid is at rest, the pressure gradient is governed by:

| $\frac{\partial p}{\partial x}=-\rho_{nf}\text{ g}.$ | (A4) |
| --- | --- |

where g is the acceleration caused by gravity and $\rho_{nf}$ is the density of the nanofluid. The downward pressure in the upper $x$-direction (aligned with gravity) is indicated by the negative sign. Standard free convection models are compatible with this hydrostatic assumption.

1. **Substitution into the Pressure Work Term**

The pressure gradient $\partial p/\partial x=-\rho_{nf}\text{ g}$ may be substituted into the pressure work term to produce

| $\beta_{nf}Tu\frac{\partial p}{\partial x}=\beta_{nf}Tu\left( -\rho_{nf}\text{ g} \right)=-\beta_{nf}\rho_{nf}\text{g }Tu\text{ }.$ | (A5) |
| --- | --- |

This factor, which represents the work done by pressure forces owing to thermal expansion in the presence of a gravitational field, is included in the energy equation (3) as $\beta_{nf}Tu \partial p/\partial x$.
